# Sesamolin Alleviates Nonalcoholic Fatty Liver Disease through Modulating Gut Microbiota and Metabolites in High-Fat and High-Fructose Diet-Fed Mice

**DOI:** 10.3390/ijms232213853

**Published:** 2022-11-10

**Authors:** Jing Yu, Hao Sun, Yang Yang, Yaping Yan

**Affiliations:** National Engineering Laboratory for Resource Development of Endangered Crude Drugs in Northwest of China, Key Laboratory of the Ministry of Education for Medicinal Resources and Natural Pharmaceutical Chemistry, College of Life Sciences, Shaanxi Normal University, Xi’an 710119, China

**Keywords:** sesamolin, nonalcoholic fatty liver disease, gut microbiota, metabonomics, correlations analysis

## Abstract

Nonalcoholic fatty liver disease (NAFLD) has become a major public health problem. The effects of sesamolin on obesity-associated NAFLD and its possible mechanism are still poorly understood. The present study investigated the effects of sesamolin on NAFLD and changes in gut microbiota and serum metabolites in high-fat and high-fructose (HF-HF) diet-fed mice. Mice with NAFLD were treated with or without sesamolin. Sesamolin effectively suppressed obesity-associated metabolic disorder, attenuated hepatic steatosis and the infiltration of inflammatory cells, and decreased levels of hepatic proinflammatory cytokines. Sesamolin also altered the composition of gut microbiota at the genus level. Additionally, differential serum metabolite biomarkers identified in an untargeted metabolomics analysis showed that sesamolin changed the levels of metabolites and influenced metabolomics pathways including caffeine metabolism, steroid hormone biosynthesis, and cysteine and methionine metabolism. Changes in metabolite biomarkers and the abundances of *Faecalibaculum*, *Lachnoclostridium*, *Mucispirillum*, *Allobaculum*, and *Bacteroides* are highly correlated with those factors involved in the progression of NAFLD. These results are important in deciphering new mechanisms by which changes in bacteria and metabolites in sesamolin treatment might be associated with the alleviation of obesity-associated NAFLD in HF-HF diet-fed mice. Thus, sesamolin may be a potential compound for obesity-associated NAFLD treatment.

## 1. Introduction

Nonalcoholic fatty liver disease (NAFLD) is a chronic liver disorder characterized by excessive fat deposition in liver cells. Clinically, with the progression of the disease, it can exhibit different pathological features in liver tissue, including changes from simple hepatic steatosis to steatohepatitis, cirrhosis, and hepatocellular carcinoma [1]. Although the pathogenic mechanisms of NAFLD are still not fully understood, it is well known that the development and progression of NAFLD is closely associated with obesity and metabolic syndrome. In recent years, due to the high prevalence of obesity and metabolic syndrome [2], the current global prevalence of NAFLD is increasing, especially in China, South America, and Europe. More worryingly, it is estimated that this prevalence will be more than 30% by 2030 [3]. An important reason for this rapid increase is associated with changes in our nutritional structure, such as an increase in the consumption of saturated fat and fructose, and a lower intake of dietary fiber and omega-3 [4,5,6,7], resulting in alterations in gut microbiota, thus leading to variations in absorption and metabolism of nutrients, and further causing the development and progression of obesity-associated NAFLD [8,9,10,11]. Since there is a gradually increasing tendency of NAFLD prevalence, there is serious concern that obesity-associated NAFLD presents a significant therapeutic challenge.

Sesame seeds (*Sesamum indicum* L.) and their oil are popular and frequently used in food cooking all over the world because they are a very good source of carbohydrates, protein, fat, microelements, and dietary fiber [12,13,14]. In addition to these nutrients, sesame seeds contain a large number of lignans [15,16], which have been proven to have numerous beneficial effects in animal experiments, such as antioxidant, anti-inflammatory, and anti-obesity effects, and improvement of the imbalance of gut microbiota [17,18,19]. Sesamolin is one of the important lignans in sesame seeds and their oil [15,16]. It can scavenge ROS and protect BV-2 cells from hypoxia-induced cell death [20], exert anti-proliferative and apoptotic effects on human colorectal cancer cells [21], enhance NK cell lysis activity on Burkitt lymphoma cells [22], and inhibit the growth of human leukemia HL-60 cells and proliferation in human lymphoid leukemia Molt 4B cells [23,24]. Moreover, sesamolin promotes cytolysis and migration activity of natural killer cells [25] and possesses neuroprotective effects against hypoxia or brain damage [26]. Additionally, sesamolin inhibits endogenous lipid peroxidation as well as oxidative DNA damage in rat liver and kidney [27]. Although studies on humans and animals have indicated that sesame oil has a strong protective effect against NAFLD [28,29,30], the effects of sesamolin on obesity-associated NAFLD and changes in gut microbiota and serum metabolites are still poorly understood.

In the present study, the preventive and therapeutic effects of sesamolin against obesity-associated NAFLD were explored in high-fat and high-fructose (HF-HF) diet-fed mice. Importantly, we attentively investigated changes in the gut microbiota and serum metabolites involved in sesamolin against NAFLD in mice. The results from this study may contribute to further understanding the role of sesamolin consumption in relieving obesity-associated NAFLD in HF-HF diet-fed mice.

## 2. Results

### 2.1. Sesamolin Suppresses Obesity and Metabolic Disorders in HF-HF Diet-Fed Mice

**Figure 1:**
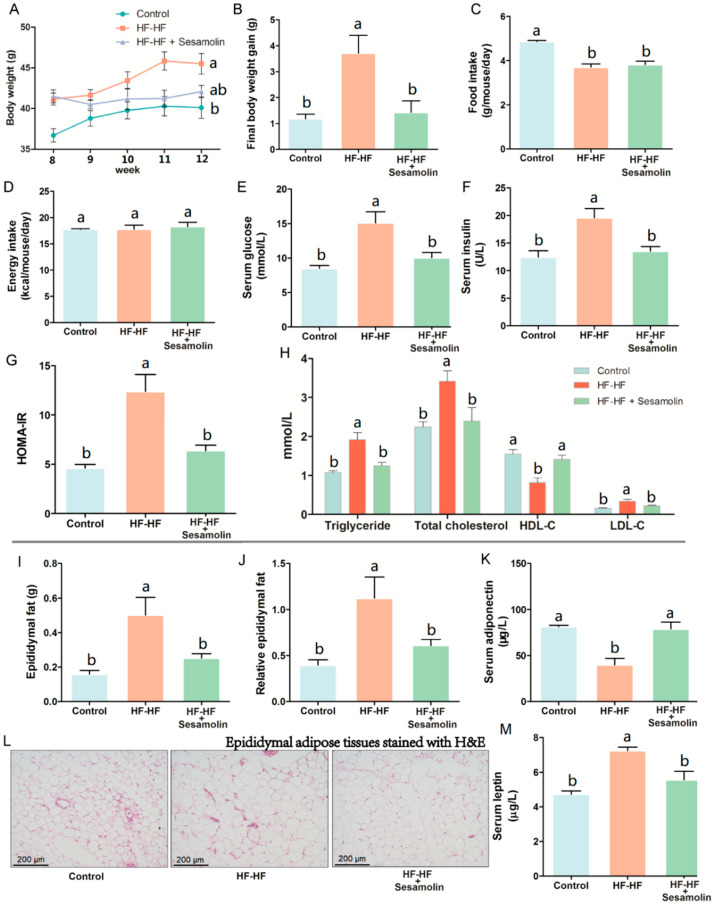
Sesamolin suppresses obesity and metabolic disorders in HF-HF diet-fed mice. (**A**) Changes in average body weight from week 9 to week 12. (**B**) Final body weight gain. (**C**) Average amount of food intake. (**D**) Average energy intake. Serum levels of (**E**) glucose, (**F**) insulin, and (**G**) HOMA-IR. (**H**) Serum levels of triglyceride, total cholesterol, HDL-C, and LDL-C. (**I**) Epididymal fat weight. (**J**) Relative epididymal fat weight. (**K**) Serum levels of adiponectin. (**L**) Epididymal adipose tissues stained with H&E. (**M**) Serum levels of leptin. Results are presented as mean ± SEM (*n* = 8). Different letters express significant differences, *p* < 0.05.

As shown in Figure 1A, the HF-HF diet caused an obvious increase in body weight at the end of week 8 compared with mice in the control group, and the body weight of mice in the HF-HF group and sesamolin-treated group rapidly increased from week 9 to week 12, but there was no significance between these two groups at the end of the experiment. Notably, compared with mice in the HF-HF group, the body weight gain of mice in the sesamolin-treated group decreased significantly at the end of the experiment (Figure 1B). Moreover, the food intake in the HF-HF group and sesamolin-treated group significantly decreased compared with mice in the control group (Figure 1C), whereas there were no significant differences in energy intake among all groups (Figure 1D). These results suggest that sesamolin suppressed obesity. In addition, long-term intake of HF-HF diet increased levels of serum triglyceride, glucose, insulin, HOMA-IR, total cholesterol, HDL-C, and LDL-C (Figure 1E–H), whereas sesamolin significantly decreased these parameters (Figure 1E–H). Additionally, overconsumption of the HF-HF diet significantly increased the epididymal fat weight, relative epididymal fat weight, and epididymal fat pad volume, along with a low level of serum adiponectin and a high level of serum leptin (Figure 1I–M). However, sesamolin treatment significantly improved these alterations (Figure 1I–M). All the above results indicate that sesamolin suppresses obesity and metabolic disorders in HF-HF diet-fed mice.

### 2.2. Sesamolin Alleviates Endotoxemia and Systemic Inflammation in HF-HF Diet-Fed Mice

**Figure 2:**
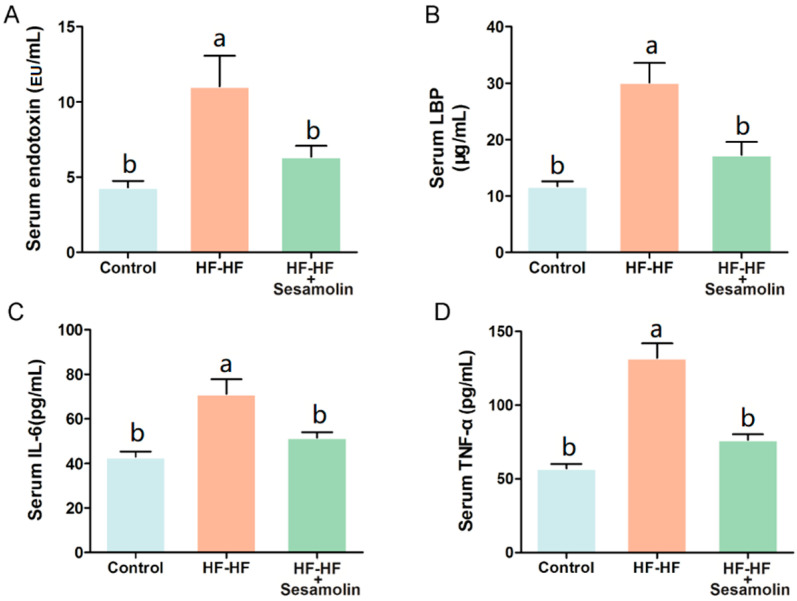
Sesamolin alleviates endotoxemia and systemic inflammation in HF-HF diet-fed mice. Serum levels of (**A**) endotoxin, (**B**) LBP, (**C**) IL-6, and (**D**) TNF-α. Results are presented as mean ± SEM (*n* = 8). Different letters express significant differences, *p* < 0.05.

At the end of the experiment, excessive consumption of an HF-HF diet significantly increased levels of serum endotoxin, LBP, IL-6, and TNF-α (Figure 2A–D). However, sesamolin treatment significantly decreased the levels of these risk factors (Figure 2A–D). Thus, sesamolin alleviates endotoxemia and systemic inflammation in HF-HF diet-fed mice.

### 2.3. Sesamolin Decreases Hepatic Steatosis and Inflammation in HF-HF Diet-Fed Mice

**Figure 3:**
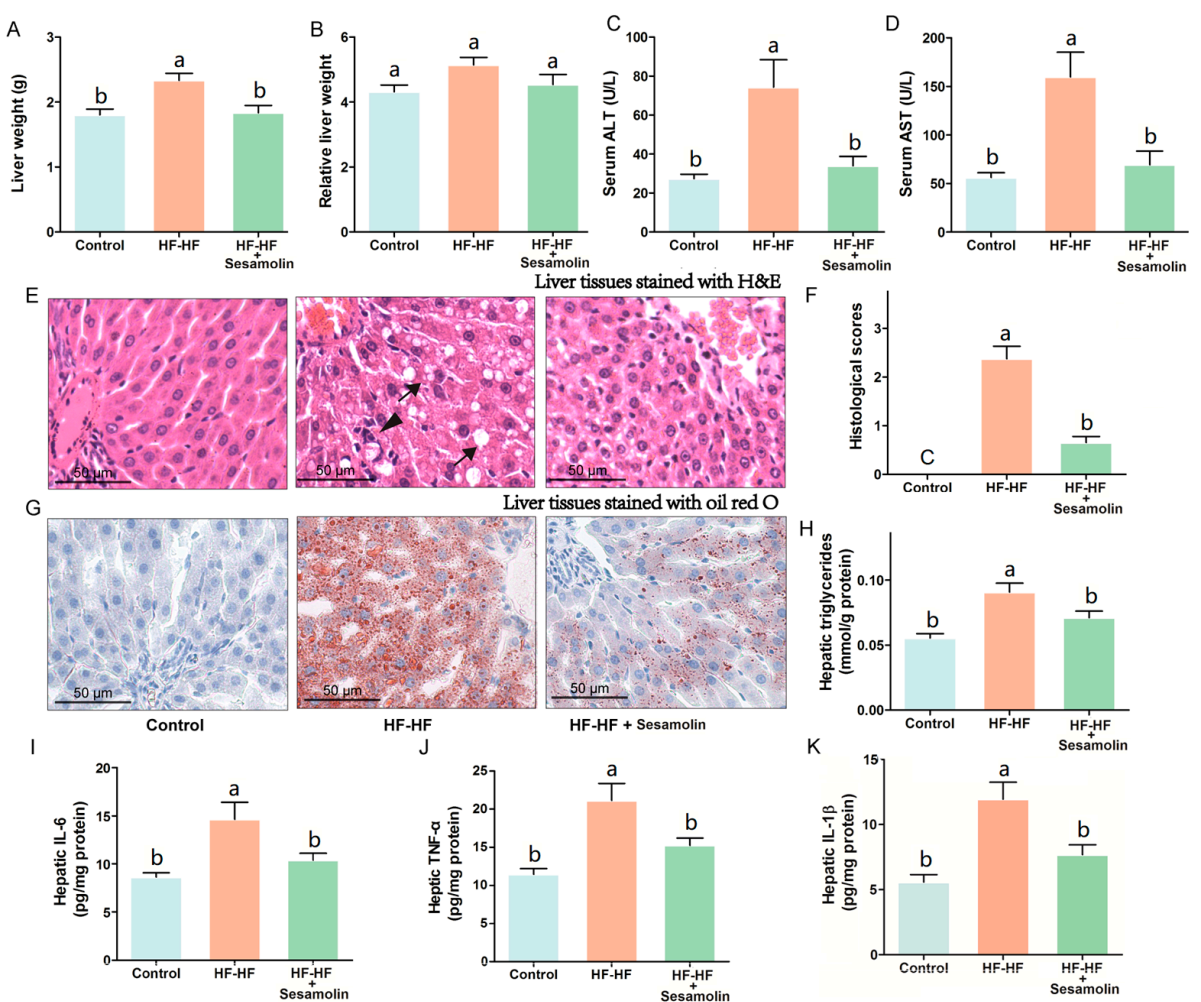
Sesamolin alleviates hepatic steatosis and inflammation progression in HF-HF diet-fed mice. (**A**) Liver weight, and (**B**) relative liver weight. Serum levels of (**C**) ALT and (**D**) AST. (**E**) Representative images of liver tissues stained with H&E: ↓, ballooning degeneration of hepatocyte, and foam cell; ▼, inflammatory cells within the hepatic lobules. (**F**) Histological scores of liver stained with H&E. (**G**) Representative images of liver tissues stained with oil red O, and (**H**) hepatic triglycerides level. (**I**–**K**) Hepatic levels of IL-6, TNF-α, and IL-1β. Results are presented as mean ± SEM (*n* = 8). Different letters express significant differences, *p* < 0.05.

To investigate the effects of sesamolin on liver damage caused by an HF-HF diet, we calculated liver weight and relative liver weight and evaluated levels of serum AST and ALT, hepatic proinflammatory response, and the changes in liver tissues stained with H&E and oil red O (Figure 3). The liver weight and relative liver weight together with serum AST and ALT levels in mice from the HF-HF group significantly increased, whereas the sesamolin-treated mice showed decreases in these indexes compared to the HF-HF group (Figure 3A–D). Additionally, HF-HF diet-fed mice without treatment presented bad distinct, widespread ballooning degeneration of hepatocytes, and a considerable amount of foamy cells, as well as inflammatory cell infiltration in hepatic lobules (Figure 3E,F). In contrast, the liver sections from the sesamolin-treated mice showed obvious improvement, as can be seen by the reductions of these alterations (Figure 3E,F). Moreover, liver tissues from mice in the HF-HF group stained with oil red O showed serious fat accumulation in hepatocytes, and higher levels of hepatic triglycerides (Figure 3G,H). However, sesamolin treatment obviously alleviated hepatic fat accumulation compared with mice in the HF-HF group (Figure 3G,H). In addition, the HF-HF diet significantly increased hepatic IL-6, TNF-α, and IL-1β levels in mice from the HF-HF group, whereas sesamolin treatment significantly decreased these inflammatory cytokines (Figure 3I–K). All the above results suggest that sesamolin decreases hepatic steatosis and inflammation progression in HF-HF diet-fed mice.

### 2.4. Sesamolin Alters the Gut Microbiota Composition in HF-HF Diet-Fed Mice

**Figure 4:**
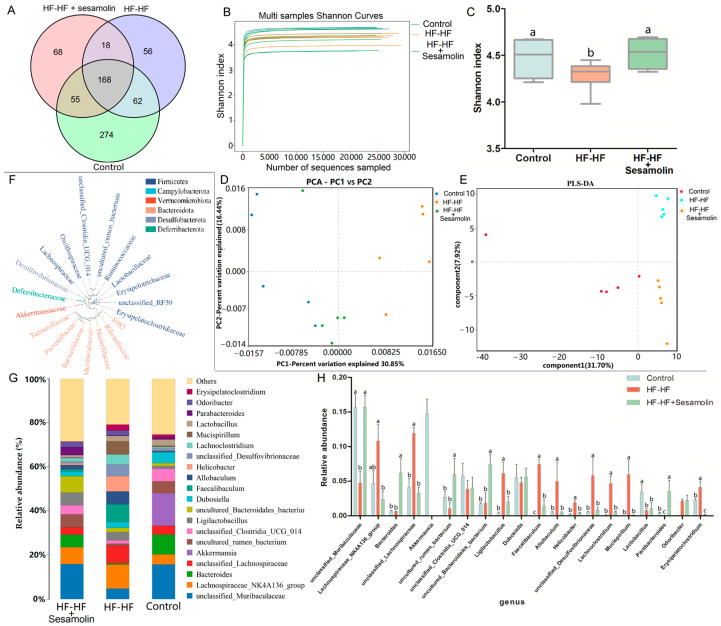
Changes in the taxonomic composition of microbial communities at the genus level. (**A**) Venn diagram showing the unique and shared bacterial genera in the different groups, (**B**) Shannon curves, (**C**) Shannon index, (**D**) PCA score plot, (**E**) PLS-DA score plot. (**F**) Phylogenetic tree showing the relationship of bacterial genera to the phylum. (**G**) Histogram showing the relative abundance of bacterial genera in the top 20, and (**H**) significantly different bacterial genera. Different letters express significant differences, *p* < 0.05.

In order to investigate the effects of sesamolin on the gut microbiota composition in HF-HF diet-fed mice, fecal samples were used for high-throughput sequencing of the V3~V4 regions of the 16S rRNA gene. After removing unqualified sequences, a total of 15,055 operational taxonomic units (OTUs) were obtained using 97% as a homology cut-off value from all samples with an average of 386 OTUs per sample. The Venn diagram shows the unique and shared bacterial genera in the different groups, where an obvious difference was exhibited among the three groups (Figure 4A). The Shannon curves of each feces sample in each group tended to be flat, indicating that the sequencing depth covered most of the diversity, and the data could be used for further analysis (Figure 4B). Additionally, the Shannon index was reduced significantly in the HF-HF diet-fed mice, suggesting that the HF-HF diet-induced lower microbiota community diversity, whereas sesamolin treatment reversed the changes (Figure 4C).

In order to evaluate the effects of sesamolin on gut microbial structure, principal component analysis (PCA) and partial least squares discrimination analysis (PLS-DA) based on unweighted UniFrac distance were carried out to reveal the differences in the gut microbial structures. PCA and PLS-DA analysis displayed distinct clustering of microbiotic composition, and multivariate analysis of variance indicated the significant separation of gut microbiota between the control group and the HF-HF group, and the HF-HF group and the sesamolin-treated group, indicating that sesamolin treatment altered gut microbial structure in HF-HF diet-fed mice (Figure 4D,E).

**Figure 5:**
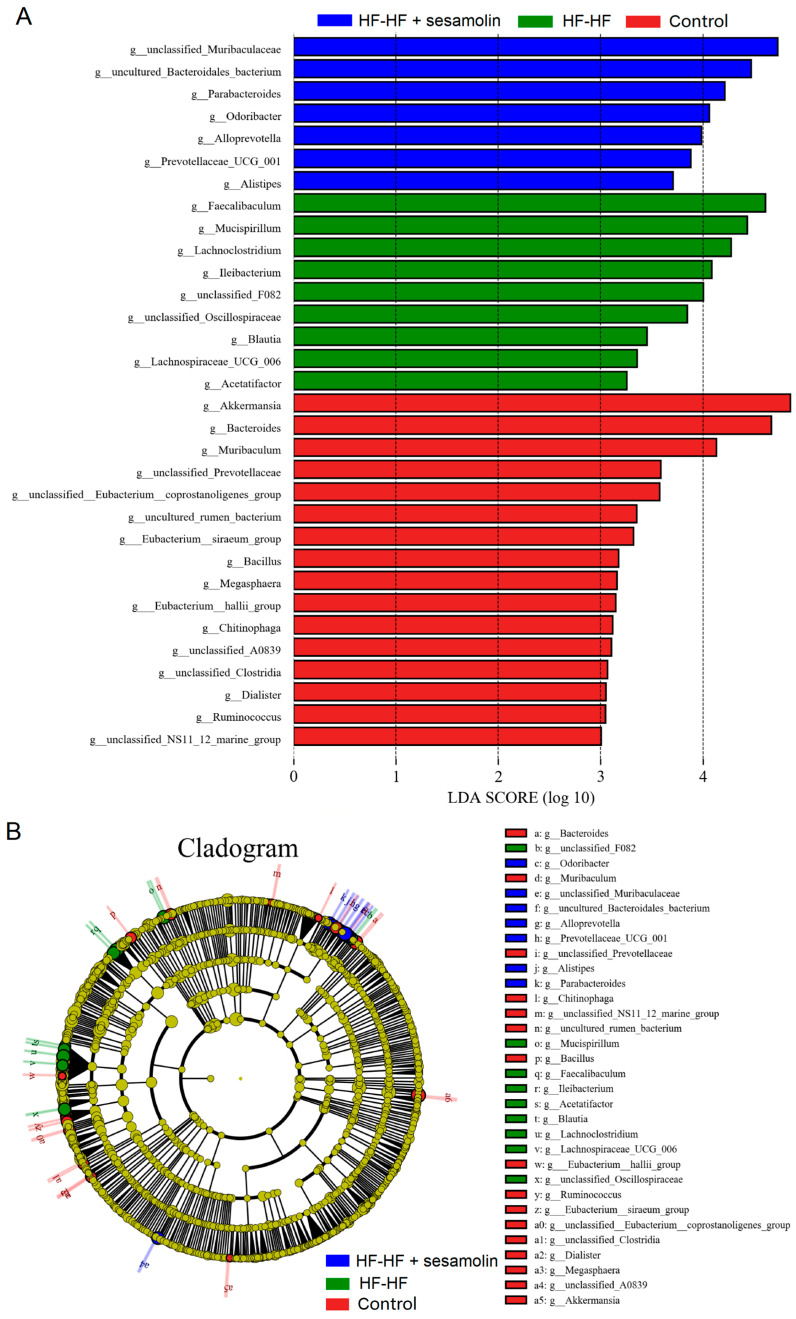
LEfSe analysis of gut microbial composition at the genus level. (**A**) Histogram of the LDA scores computed for differentially abundant bacterial taxa. (**B**) The Cladogram of taxonomic distribution in the different groups (LDA score, >3.5). Different letters express significant differences, *p* < 0.05.

In order to assess specific changes in the gut microbiota, the relative abundances of the predominant taxa were analyzed at the genus level. As shown in Figure 4F,G, the phylogenetic tree shows the relationship of bacterial genera to the phylum, and the histogram exhibits the relative abundance of bacterial genera in the top 20. At the genus level, compared with mice in the control group, the relative abundance of unclassified_*Muribaculaceae* decreased significantly, while the relative abundance of *Helicobacter*, *unclassified_Lachnospiraceae*, *Faecalibaculum*, *Lachnoclostridium*, *Allobaculum*, unclassified_*Desulfovibrionaceae*, and *Mucispirillum* increased significantly (Figure 4G,H). However, compared to mice in the HF-HF group, the relative abundance of *Bacteroides*, unclassified_*Muribaculaceae*, *and Parabacteroides* increased significantly, while the relative abundance of *Lachnoclostridium*, *Faecalibaculum*, uncultured_*Bacteroidales*_bacterium, unclassified_*Lachnospiraceae*, *Allobaculum*, *Helicobacter*, unclassified_*Desulfovibrionaceae*, and *Mucispirillum* decreased significantly in the sesamolin-treated group (Figure 4G,H). To further reveal the effect of sesamolin on gut microbial composition, LEfSe was used to identify specific taxa (Figure 5A,B). Twenty taxa were lower, and nine were higher in the HF-HF group compared with mice in the control group, whereas nine taxa were higher, and four were lower in the sesamolin-treated group than in the HF-HF group. At the genus level, *Ileibacterium*, *Faecalibaculum*, *Lachnoclostridium*, unclassified_*Oscillospiraceae*, unclassified_*F082*, and *Mucispirillum* were the main dominant bacteria in the HF-HF group (Figure 5A,B). Whereas *Odoribacter*, *Alistipes*, *Parabacteroides*, *Prevotellaceae*_UCG_001, *Alloprevotella*, unclassified_*Muribaculaceae*, and uncultured_*Bacteroidales*_bacterium were the dominant bacteria in the sesamolin-treated mice (Figure 5A,B). These data indicate that sesamolin effectively improves gut microbial structure at the genus level in HF-HF diet-fed mice.

### 2.5. Sesamolin Alters Serum Metabolome in HF-HF Diet-Fed Mice

**Figure 6:**
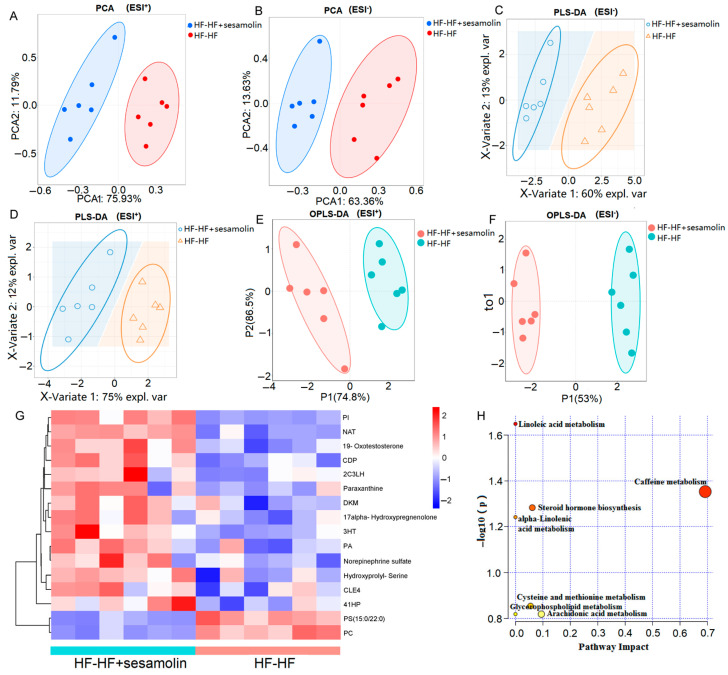
Untargeted metabolomics of the serum metabolomes in the HF-HF group and the HF-HF + sesamolin group. (**A**,**B**) PCA score plot; (**C**,**D**) PLS-DA score plot; (**E**,**F**) OPLS-DA score plot; (**G**) Heatmap of the significantly different metabolites (VIP > 1.0, and *p* < 0.05 with 95% confidence intervals): (PA(15:0/18:4(6Z,9Z,12Z,15Z))) PA, (2-(3-Carboxy-3-(methylammonio)propyl)-L-histidine) 2C3LH, (N-Arachidonoyl Threonine) NAT, (PI(22:4(10Z,13Z,16Z,19Z)/6keto-PGF1alpha)) PI, ((4Z,10Z,12E)-3-Hydroxytetradeca-4,10,12-trienoylcarnitine) 3HT, (20-COOH-leukotriene E4) CLE4, (PC(16:1(9Z)/22:6(4Z,7Z,10Z,13Z,16Z,19Z))[U]) PC, (CDP-DG(a-21:0/PGF1alpha)) CDP, (1,2-Dihydroxy-3-keto-5-methylthiopentene) DKM, and ((4Z,7Z,10E,12E,16Z)-18-(3-Ethylcycloprop-1-en-1-yl)-14-hydroxyoctadeca-4,7,10,12,16-pentaenoylcarnitine) 41HP. (**H**) The metabolic pathway impact prediction of significantly different metabolites based on the KEGG online database.

To explore the effects of sesamolin on serum metabolic response, serum metabolic profiles from the HF-HF group and the sesamolin-treated group were carried out using liquid chromatography–mass spectrometry (LC-MS). A total of 16,807 peaks were detected (the number of ESI^+^ and ESI^−^ ions was 7581 and 8956, respectively), and a total of 4060 metabolites were identified (the number of ESI^+^ and ESI^−^ ions was 2260 and 1800, respectively). The PCA results showed divergent trends in the serum metabolic profile between the HF-HF group and the sesamolin-treated group (Figure 6A,B). The PLS-DA and OPLS-DA were used to better distinguish the metabolites between the HF-HF group and the sesamolin-treated group, and the results showed that the serum metabolic profiles were significantly different between these two groups, suggesting that sesamolin treatment led to obvious changes in the serum metabolome composition (Figure 6C–F). According to a variable value set at a variable importance in projection (VIP) (VIP > 1 and *p*-value < 0.05) in a Wilcoxon rank-sum test, a total of 16 serum metabolite biomarkers were found as significant alterations by sesamolin treatment, in which 14 metabolites were upregulated and 2 metabolites were downregulated in the sesamolin-treated group, compared with the HF-HF group (Figure 6G). The metabolic pathways of these metabolite biomarkers were analyzed with MetaboAnalyst, and a total of seven metabolic pathways were associated with the metabolites. Among these metabolic pathways, according to the *p* values of <0.05 and impact values of >0, the most relevant pathways were caffeine metabolism, steroid hormone biosynthesis, and cysteine and methionine metabolism (Figure 6H). Taken together, these data reveal that sesamolin treatment significantly improves the serum metabolic profile and metabolic pathways.

### 2.6. Correlation Analysis

All the above-described results show that sesamolin treatment leads to alterations in gut microbiota, improves serum metabolome alterations and metabolic disorders, alleviates endotoxemia and systemic inflammation, and decreases hepatic steatosis and inflammation in HF-HF diet-fed mice. In order to investigate whether these parameters in serum and liver were correlated with gut microbiota and metabolite biomarkers, we performed a Pearson’s correlation analysis. We selected the 20 most abundant genera and relative parameters from the HF-HF group and the sesamolin-treated group for further correlation assessment.

**Figure 7:**
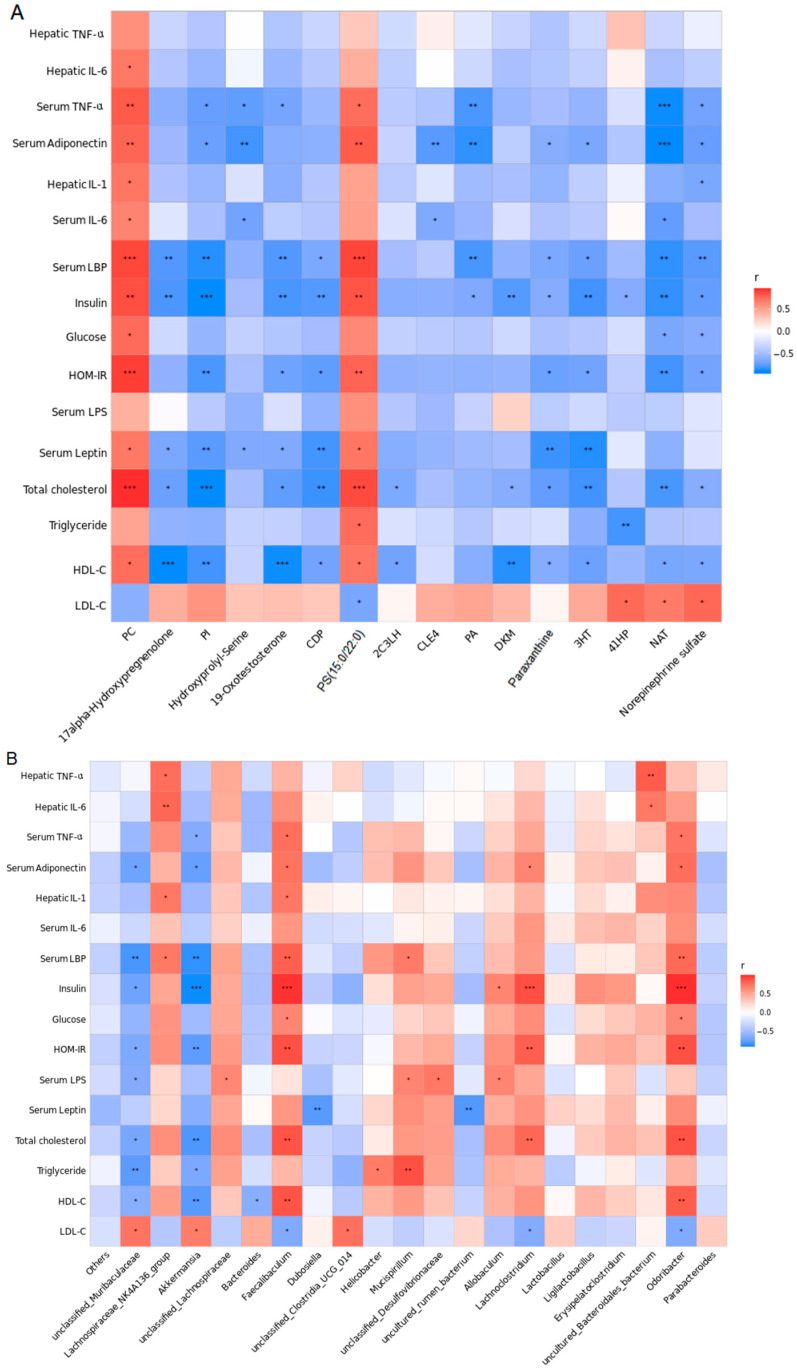
Results of correlation analysis. (**A**) Correlation heat map between the 16 serum metabolite biomarkers and risk factors of NAFLD from the HF-HF and HF-HF + sesamolin groups. (**B**) Correlation heat map between the 20 most dominant genera and risk factors of NAFLD from the HF-HF and HF-HF + sesamolin groups. * p < 0.05; ** p < 0.01; *** p < 0.001.

The correlations between the gut microbiota genera and serum metabolite biomarkers are shown in Figure 7A, where metabolites were more highly correlated with serum endotoxemia (endotoxin and LBP), body proinflammatory response cytokines (IL-6 and TNF-α), adipocytokines (adiponectin and leptin), and hepatic proinflammatory cytokines (IL-6, TNF-α, IL-1β). Moreover, as shown in Figure 7B, serum endotoxemia (endotoxin and LBP), body proinflammatory response cytokines (IL-6 and TNF-α), adipocytokines (adiponectin and leptin), and hepatic proinflammatory cytokines (IL-6, TNF-α, IL-1β) were closely linked to *Dubosiella*, *Akkermansia*, *Allobaculum*, *Helicobacter*, *Odoribacter*, *Faecalibaculum*, *Lachnoclostridium*, *Lachnospiraceae*_NK4A136_group, *Bacteroides*, unclassified_*Lachnospiraceae*, *Mucispirillum*, unclassified_*Clostridia*_UCG_014, uncultured_*rumen*_bacterium, unclassified_*Muribaculaceae*, unclassified_*Desulfovibrionaceae*, and uncultured_*Bacteroidales*_bacterium.

## 3. Discussion

Excessive consumption of a high-fat and/or high-fructose diet is considered a key reason for the formation and progression of NAFLD. Previously, sesame oil has been proven to have a strong protective effect against NAFLD [20,21,22]. Sesamolin is one of the major lignans in sesame oil [15]. Whether sesamolin has effects on obesity-associated NAFLD is less understood. In this study, HF-HF diet feeding caused the formation and progression of obesity-associated NAFLD in mice, such as excessive body weight gain, serious hepatic steatosis, and infiltration of inflammatory cells, as well as alterations in endotoxemia (endotoxin and LBP), body proinflammatory cytokines (IL-6 and TNF-α), adipocytokines (adiponectin and leptin), and hepatic proinflammatory cytokines (IL-6, TNF-α, IL-1β). However, sesamolin treatment at a dose of 60 mg/kg inhibited obesity, and alleviated hepatic steatosis and inflammatory response, along with the improvement of these indexes in HF-HF diet-fed mice. Previous studies have suggested that the recommended dose for sesame oil in the treatment of fatty liver in humans is 30 g per day [28,29], while the content of total lignans (mainly including sesamin and sesamolin) in sesame oil ranges from 4.86 to 10.66 mg/g [15]. Thus, the dose for the total lignans in this study [28,29] should be 2.43~5.33 mg/kg (calculated by the body weight of 60 kg). According to dose conversion between mice and humans [31], the dose of sesamolin for humans based on our study is close to the dosage of the total lignans in sesame oil in the treatment of fatty liver [28,29]. Naturally, whether this quantity is an effective dose for humans needs to be further investigated in clinical practice.

The formation and progression of NAFLD suffers from “multiple parallel hits”, mainly including gut-derived signals and adipose tissue-derived signals [32]. Endotoxin, one of the main gut-derived signal molecules, is produced in gut microbiota, where it has been demonstrated that intake of a high-fat or a high-carbohydrate diet both in humans and in animals could lead to an increase in blood endotoxin concentrations. Increased serum endotoxin concentrations might not only lead to systemic inflammation but also exacerbate obesity itself, leading to NAFLD progression. In addition, adipose tissue-derived signals are a risk factor for NAFLD progression since they concern an immune organ with the capacity to produce a wide range of adipocytokines and cytokines, such as adiponectin, leptin, TNF-α, and IL-6 [32]. Although changes in these adipocytokines (adiponectin and leptin) and cytokines (TNF-α and IL-6) may have local effects on adipose tissue physiology, they also can create effects targeting hepatic fat accumulation and inflammation progression [32]. In our study, long-term intake of an HF-HF diet induced an increase in levels of serum endotoxin, LBP, IL-6, TNF-α, and leptin, and a decrease in serum adiponectin level, a result that is similar to previous reports [33,34]. In contrast, sesamolin treatment decreased these serum biochemical parameters, suggesting that the result that sesamolin could inhibit obesity and regulate lipid metabolism was associated with its effects on the regulation of these risk parameters.

Many studies have indicated that a high-fat and/or high-fructose diet could induce intestinal microflora dysbiosis in patients with NAFLD or animal models. Although no definitive bacterial biomarkers associated with NAFLD were found, numerous studies have indicated the same tendency in some specific genera [35]. In the present study, an HF-HF diet induced significant changes in gut microbiota, whereas sesamolin treatment reversed these alterations in HF-HF diet-fed mice. At the genus level, long-term intake of an HF-HF diet increased the relative abundance of *Helicobacter*, *Faecalibaculum*, *Lachnoclostridium*, *Allobaculum*, *Odoribacter*, and *Mucispirillum*. It has been suggested that *Helicobacter* [36] and *Lachnoclostridium* [37] might lead to the pathogenesis of NAFLD. *Faecalibaculum* [38] and *Mucispirillum* [39] were closely linked to obesity, oxidative stress, or gut inflammation, and *Allobaculum* [40] and *Odoribacter* [41] increased the risk of diabetes progression, in which these risk factors might be all the main reason for the exacerbation of NAFLD. However, sesamolin-treated mice showed a lower relative abundance of *Lachnoclostridium*, *Faecalibaculum*, *Allobaculum*, *Helicobacter*, and *Mucispirillum*. Additionally, some previous studies demonstrated that a lower relative abundance of *Bacteroides* and *Parabacteroides* presented in NAFLD patients [42] or animals [43,44]. *Parabacteroides* was mostly reported as a beneficial bacteria defending against atherosclerosis and NAFLD [45], and *Alistipes* [46] may contribute to improving liver fibrosis and cardiovascular disease. Moreover, the abundance of the family *Prevotellaceae* is higher in healthy subjects than NAFLD patients [47], and *Alloprevotella* proved to be a beneficial bacteria in one study [48] in which the alterations in *Alloprevotella* and *Prevotellaceae*_UCG_001 (family *Prevotellaceae)* were also observed in animal experiments [49,50]. Notably, the relative abundance of *Bacteroides* and *Parabacteroides* was increased, *and Alistipes*, *Prevotellaceae*_UCG_001, and *Alloprevotella* were the main dominant bacteria in sesamolin-treated mice. The above results indicate that sesamolin treatment alleviates NAFLD progression in association with the improvement of intestinal flora. As well as for the above-mentioned bacteria, including *Bacteroides*, *Parabacteroides*, and *Prevotellaceae*, studies on humans also indicated that *Helicobacter*, *Lachnoclostridium*, *Faecalibaculum*, and *Lachnoclostridium* are relevant microbiota in patients with NAFLD [51]. The alterations to some dominant bacteria from this study are similar to human studies, which provide some preclinical practice for clinical study. However, whether sesamolin has an effect on the modulation of these gut microbiota in people with obesity-associated NAFLD, and changes in the other bacteria observed in this study, needs further exploration.

Serum metabolic alterations are associated with NAFLD progression. In this study, excessive consumption of an HF-HF diet induced significant alterations in the serum metabolic profiles, whereas sesamolin treatment reversed these changes. The Wilcoxon rank-sum test based on the VIP value exposed the significantly different biomarkers between the sesamolin-treated group and the HF-HF group, in which previous studies have indicated that paraxanthine exerted an anti-obesity effect [52], and PC showed a higher level [53] in high-fat diet-fed animals and is in accordance with our study. Moreover, 19-Oxotestosterone was indicated at a lower level in patients with alcoholic liver disease [54,55], and a similar result was presented in this study, suggesting that a lower 19-Oxotestosterone metabolism might also be linked to NAFLD progression. In contrast, sesamolin treatment that increased the relative intensity of 19-Oxotestosterone contributes to the improvement of obesity-associated NAFLD. Certainly, further research is needed to confirm the roles of 19-Oxotestosterone and its metabolic pathways in NAFLD progression. Additionally, the present study further found lower levels of 2C3LH, N-Arachidonoyl threonine, 3HT, CDP, 41HP, PI, CLE4, Norepinephrine sulfate, 17alpha-Hydroxypregnenolone, DKM, and PA, and higher levels of PS (15:0/22:0) in the serum, which are directly correlated with those risk factors in the progression of NAFLD in HF-HF diet-fed mice. However, sesamolin treatment was contrary to the results of the HF-HF-fed group, corresponding to the alleviation of obesity-associated NAFLD. Interestingly, it is well known that drug intervention can potentially influence the metabolism of nutrients via effects on appetite, absorption, gastrointestinal motility, hepatic metabolism, and gut microbiota [56]. The result from metabolites suggests that sesamolin treatment might influence the absorption and metabolism of some nutrients in the HF-HF diet-fed mice, and thereby, the improvement in obesity-associated NAFLD might be linked to the effect of sesamolin on the absorption and metabolism of these nutrients from the HF-HF diet in mice.

Increasing evidence has revealed that the correlation between serum metabolic alterations and gut microbiota dysbiosis helps explain the possible mechanisms of obesity-associated NAFLD progression [50,57]. At the genus level, a significant correlation between the gut microbiota and risk factors of NAFLD is shown in this study. Interestingly, we found that the main dominant bacteria in the HF-HF group, that is, *Faecalibaculum*, *Lachnoclostridium*, and *Mucispirillum*, present a significantly negative correlation with those beneficial factors, while *Helicobacter* shows a significantly positive correlation with those high-risk factors of NAFLD. In addition, the main dominant bacteria in sesamolin-treated mice, that is, *Allobaculum*, and *Bacteroides*, showed a significantly positive correlation with those beneficial factors, and a significantly negative correlation with those high-risk factors of NAFLD. The correlation analysis reveals that *Bacteroides*, *Faecalibaculum*, *Lachnoclostridium*, *Mucispirillum*, and *Allobaculum* might influence multi-parameters, and each of these parameters is affected by multi-bacteria genera, suggesting that the alterations of NAFLD are more highly correlated with changes in bacteria genera in HF-HF diet-fed mice.

In conclusion, our findings provide relevant insight into the potential role of sesamolin against NAFLD progression in HF-HF diet-fed mice. In this study, we demonstrated that sesamolin treatment improved intestinal flora at the genus level in HF-HF diet-fed mice. Additionally, sesamolin treatment altered the serum metabolic phenotype of NAFLD mice. We also identified possible correlations between bacterial functionality and related parameters. These results are essential to deciphering new mechanisms by which changes between bacteria and metabolites in sesamolin treatment might be associated with the alleviation of obesity-associated NAFLD. These findings suggest the possibility that sesamolin may be a potential compound for obesity-associated NAFLD treatment.

## 4. Experimental Section

### 4.1. Animal Experimental Design and Sample Collection

C57BL/6J mice (9~10 weeks old, 23 ± 4 g) were provided by Beijing Vital River Laboratory Animal Technology Co., Ltd. (Beijing, China), and housed at 25 ± 3 °C, 30–70% humidity and normal light/dark (12 h/12 h) cycle conditions. All animal experimental procedures were carried out in accordance with the National Institutes of Health guide for the care and use of laboratory animals (NIH Publications No. 8023, revised 1978). Use of the mice was reviewed and approved by both Shaanxi Normal University and the local animal ethics committee (SYXK(SHAN)2021-003). Sesamolin (Pubchem CID: 101746, purity ≥ 98%) was obtained from Shanghai Macklin Biochemical Co., Ltd. (Shanghai, China). Normal chow diet (3.64 kcal/g, 10 kcal% fat) and HF-HF diet (5.12 kcal/g, 60 kcal% high-fat diet supplemented with 10% fructose) were provided by SYSE Bio-tech. Co., Ltd. (Changzhou, China).

After 1 week of acclimation, all animals were weighed and randomly divided into three groups with eight animals, and housed with four mice per cage. The experiments were carried out for the 12 weeks of the experimental period with the following procedure: control group with normal chow diet, HF-HF group (mice fed with HF-HF diet without treatment), HF-HF plus sesamolin group (HF-HF + sesamolin, mice fed with HF-HF diet and orally administrated sesamolin at a dose of 60 mg/kg twice a day from week 9 to week 12, while mice in others groups received the same solvent as a placebo in this period). Food intake was noted every day, and body weight was monitored every day on week 0, and at week 8 to week 12. Fecal samples from five individual mice in each group were collected at week 12 and were stored at −80 °C for DNA sequencing. At the end of the experimental period, the animals were anesthetized and sacrificed by cervical dislocation after collecting blood samples through retro-orbital plexus under anesthesia. The epididymal fat and liver tissues were collected and weighed, and used for pathological study and biochemical analysis. Blood was centrifuged at 5000 rpm (4 °C) to obtain serum for biochemical parameter analyses.

### 4.2. Measurement of Serum Biochemical Parameters

Serum triglycerides, glucose, total cholesterol, high-density lipoprotein cholesterol (HDL-C), low-density lipoprotein cholesterol (LDL-C), aspartate aminotransferase (AST), and aspartate transaminase (ALT) were detected using commercially available detection kits (Boxbio, Beijing, China). Serum endotoxin, insulin, adiponectin, leptin, lipopolysaccharide-binding protein (LBP), tumor necrosis factor-α (TNF-α), and interleukin-6 (IL-6) were analyzed using ELISA kits (Boxbio, Beijing, China). HOMA-IR = insulin (U/L) × glucose (mmol/L)/22.5.

### 4.3. Histological Analysis

Fresh liver tissues and epididymal fat tissues were treated with a 4% paraformaldehyde solution (pH 7.4). After 48 h, these tissues were embedded in paraffin and subsequently cut at 4 μm using a Leica RM2235 Tissue Slicer (Shanghai Jumu Medical Instrument Co., Ltd., Shanghai, China). Then, the selected tissues were mounted on clean glass slides, deparaffinized and rehydrated, and then stained with commercially available hematoxylin and eosin (H&E) staining solutions (Solarbio, Beijing, China), respectively. In addition, the severity of hepatic steatosis was assessed by oil red O: Briefly, liver tissue treated with 4% paraformaldehyde solution was embedded in an optimal cutting temperature (OCT) compound, and then liver sections (5 μm) were washed with sterile water, infiltrated with 60% isopropanol, and treated with hematoxylin followed by staining with oil red O staining solution (Solarbio, Beijing, China). The histopathological changes in liver and epididymal fat tissues were examined under a light microscope (Olympus Corporation, Tokyo, Japan). The histopathological scores in liver tissues stained with H&E were evaluated based on a previously reported method [58] and obtained by ballooning degeneration score plus inflammatory cell infiltration score: ballooning degeneration (grade 0, normal hepatocytes; grade 1, presence of clusters of hepatocytes with a rounded shape and pale cytoplasm; grade 2, at least one enlarged ballooned hepatocyte); inflammatory cell infiltration (grade 0, none; grade 1, <2 foci per lobule; grade 2, >2 foci per lobule).

### 4.4. Measurements of Hepatic TNF-α, IL-6, Interleukin- 1β (IL-1β) and Triglycerides

Liver tissues were homogenized with cold PBS buffer (100:1, mg/mL, pH 7.4). The homogenates were obtained by centrifuging at 12,000× *g* for 30 min at 4 °C and used for the determinations of hepatic TNF-α, IL-6, IL-1β, and triglycerides. Hepatic TNF-α, IL-6, and IL-1β were measured using ELISA kits (Boxbio, Beijing, China). Hepatic triglycerides were measured by a triglycerides assay kit (Solarbio, Beijing, China) according to the manufacturer’s instructions.

### 4.5. Analysis of Gut Microbiota

Fecal samples from five animals from each group were used for the analysis of gut microbiota. Microbial DNA was extracted from fecal samples using a DNA extraction kit (Tiangen Biotech Co., Ltd., Beijing, China) according to the manufacturer’s instructions. Then, DNA amplification, library construction, sequencing, and data analysis were performed. The full length of bacterial 16S rDNA genes was amplified using barcoded conserved primers 27F (5′-AGAGTTTGATCCTGGCTCAG-3′) and 1492R (5′-GGTTACCTTGTTACGACTT-3′). The amplified library was sequenced using a PacBio SMRT RS III DNA sequencing platform (Pacific Biosciences, Menlo Park, CA, USA). The sequences were filtered by PacBio circular consensus sequencing technology with cutadapt 1.9.1 software, and then related results were analyzed using Usearch, mothur (version 197 v.1.30), and QIIME2 software at BMK Cloud (accessed on 1 August 2020; www.biocloud.net).

### 4.6. Non-Targeted Metabolite Profiling Analysis

The serum samples from the HF-HF group and the HF-HF + sesamolin group were detected, and six biological replicates in each group were performed. All samples were extracted according to Li et al. described [59]. The non-targeted metabolic profiling analysis was performed as previously described [60]. Briefly, ultra-high-performance liquid chromatography coupled with high-resolution mass spectrometry (Q Exactive Focus, Thermo Scientific, Waltham, MA, USA) was used for sample analysis. Each sample was analyzed using a reversed-phase chromatography column (2.1 mm × 150 mm, 3 µm, Merck Corporation, Darmstadt, Germany) at 30 °C. The mobile phase consisted of methanol (solution A) and water (solution B), both containing 0.1% *v*/*v* formic acid. The gradient was: 0–5 min, 100% A; 5–20 min, 100% B → 0% A; 10–10.1 min, 0% A → 100% A; 20.1–25 min, 100% A, and the flow rate was 0.5 mL/min. The data were acquired in both positive (ESI^+^) and negative (ESI^−^) electrospray ionization modes. Quality control samples were injected at the beginning of the analysis. Data were obtained in centroid mode using FreeStyle 1.3 (Thermo Scientific, Waltham, MA, USA).

### 4.7. Statistical Analysis

All results are presented as the mean ± standard error of mean (SEM). The data were evaluated with SPSS 16.0 software (Chicago, IL, USA). Statistical significance was determined by one-way analysis of variance (ANOVA) followed by Tukey’s test, and significance was set at *p* < 0.05.

## Data Availability

The data that support the findings of this study are available from the corresponding author upon reasonable request.

## Abbreviation

NAFLD: nonalcoholic fatty liver disease; HF-HF: high-fat and high-fructose; HDL-C: high-density lipoprotein cholesterol; LDL-C: low-density lipoprotein cholesterol; AST: aspartate aminotransferase; ALT: aspartate transaminase; LBP: lipopolysaccharide binding protein; TNF-α: tumor necrosis factor-α; IL-6: interleukin-6; H&E: hematoxylin and eosin; HOMA-IR: homeostasis model assessment; IL-1β: interleukin-1β; SEM: standard error of mean; PCA: principal component analysis; PLS-DA: partial least squares discrimination analysis; OPLS-DA: orthogonal partial least squares-discriminant analysis.
